# Primary Care, Burnout, and Patient Safety: Way to Eliminate Avoidable Harm

**DOI:** 10.3390/ijerph191610112

**Published:** 2022-08-16

**Authors:** Yoshito Nishimura

**Affiliations:** 1 Department of General Medicine, Okayama University Graduate School of Medicine, Dentistry and Pharmaceutical Sciences, Okayama 700-8558, Japan; nishimura-yoshito@okayama-u.ac.jp; 2 Department of Medicine, John A. Burns School of Medicine, University of Hawai’i, Honolulu, HI 96813, USA

## 1. Introduction

Patient safety has been a big theme in the area of global health, as represented by the resolution of the World Health Organization (WHO) on “Global action on patient safety” in 2019 and the recently published “Global Patient Safety Action Plan 2021–2030 [1].” One of the reasons behind the attention to patient safety is the significance of preventable harm from unsafe care, estimated as having a social cost of 1–2 trillion USD a year [2]. Due to the collaborative efforts of stakeholders, including government organizations, healthcare facilities, and frontline clinicians, in-hospital patient safety issues have acquired attention over in recent years. However, patient safety in primary care or outpatient settings has been left behind, likely because we have focused on in-hospital phenomena, such as hospital-acquired infection (HAI). Despite this, a considerable amount of healthcare occurs in outpatient settings, and multiple factors contribute to patient safety issues, such as the transition of care, medication errors, burnout and physician wellness [3,4,5,6]. The WHO also estimated that 4 in 10 patients had been harmed in primary care settings. In particular, burnout and physician wellness has been noted as a crucial component of providing quality care. This article will cover the significance of patient safety in primary care settings, focusing on burnout.

## 2. Factors Related to Patient Safety in Primary Care

Multiple factors are related to patient safety in primary care or ambulatory settings. Unlike inpatient care, clinicians and patients usually only have the chance to see each other weeks or months apart [6]. Thus, it is essential to ensure that patients and caregivers are involved in their care and establish contingency plans in case of treatment failure or emergency.

Additionally, medication and diagnostic errors are common, leading to up to 4.5 million unnecessary visits per year. As medication errors incur the considerable cost of up to 42 billion USD annually [7], the WHO has recently proposed a strategic objective for its member countries to establish quantifiable national targets on medication-related harm [1]. As medication errors have various causes, including clinician-, patient-, and task-related causes, multifaceted approaches to completing a thorough medication review are essential, as well as reconciliation by frontline clinicians and pharmacists, clinician and patient education, and improvements in electronic medical records (EMR) to update and integrate medication lists from multiple sources. Diagnostic errors are related to different patient processes, such as issues with referral, patient-related factors such as no-shows and language issues, missed opportunities to follow-up on diagnostic tests, and clinicians’ interpretations of the tests [8]. Since we only have limited time in ambulatory encounters, clinicians often use system 1 thinking, an intuitive process based on their own experiences. Due to uncertainties related to ambulatory medicine, it is crucial to employ the system 2 approach and further optimizae diagnostic strategies, implantat a system enabling feedback to be provided clinicians regarding diagnostic errors, and involve patients and caregivers [9,10].

A factor that is often overlooked is burnout, a psychological status related to occupational stress characterized, according to the WHO, by feelings of energy depletion or exhaustion; increased mental distance from one’s job, or negative or cynical feelings related to one’s job; and reduced professional efficacy [11,12]. Maslach characterized symptoms of burnout as emotional exhaustion, depersonalization, and a reduction in personal accomplishment [13]. During the COVID-19 pandemic, physician burnout gained significant attention from academia and the general public, as the Pubmed search identified more than 7000 articles including the term ‘burnout’ from 2020 to April 2022. In her article, Maslach mentioned that “the twin goals of preventing burnout and building engagement are possible and necessary in today’s working world. Rather, people have to work together to make them happen. Rather, people have to work together to make them happen [14]”. Despite the importance of leadership efforts, this fact is often overlooked in primary care due to the nature of ambulatory care. What is the current situation concerning burnout in primary care and its relation to patient safety, and how could we address the issues?

## 3. Burnout in Primary Care

There is substantial evidence that burnout may negatively affect patient safety, which is multifactorial, with depersonalization and emotional exhaustion decreasing productivity, quality of care, and increasing diagnostic errors [15]. However, the burnout of healthcare workers in primary care settings failed to garner attention, even during the COVID-19 pandemic, likely due to the misconception that those providing acute inpatient medical care were more affected by the pandemic. However, that might not be true. Given the rapid implementation of telemedicine, leading to considerable changes in the primary care work environment, and the emergence of outpatient treatment options, primary care workers have also been overwhelmed by the pandemic. Additionally, moral injury, which was further accentuated during the pandemic, is a crucial component of burnout. In countries such as the US, primary care providers commonly face situations where they cannot prescribe guideline-directed, medically appropriate medications or order necessary testing, given restrictions related to patients’ insurance status. While providers are used to these challenges, the experiences may eventually lead to feelings of helplessness due to their inability to provide the best possible care. The emergence of telehealth visits could have exacerbated the issue of moral injury. While telehealth has become an essential part of healthcare, this option is not suitable for every patient and needs to be limited to specific chief complaints or the follow-up of stable chronic patients. Primary care providers faced a situation where they had no choice but to tell their patients to reschedule in-person appointments or to go to an urgent care clinic or emergency department. As a result, primary care physicians or general practitioners are known to suffer from a higher prevalence of burnout than other specialties, reaching up to 70% depending on the research [16].

How can we best address the burnout of primary care providers and improve patient safety? Much attention has been paid to individual efforts to improve resiliency or work engagement. As Maslach noted in the 1990s, burnout is not only a problem of individuals but also a problem for the practice, organization, and health system to which the providers belong. The optimization of electronic medical records to decrease unnecessary work burden, creation of a good balance between autonomy and support, and ensuring a work–life integration while securing providers’ private time could be solutions [17]. An increase in awareness of burnout among leadership is crucial.

## 4. Conclusions

The pursuit of improved patient safety in primary care involves multiple factors. One essential factor is burnout. The healthcare landscape has been dynamically changing each day since the COVID-19 pandemic. Given the spread of potentially contagious pathogens such as Monkeypox, and significant changes in healthcare policy, such as the recent overturn of Roe v. Wade by the US Supreme Court, resilience and adaptive capacities have become one of the most crucial skills for primary care providers. System- and leadership-level efforts are crucial to address issues related to patient safety, particularly burnout, given the increasing complexity of medicine.
